# Impact of maternal dietary carbohydrate intake and vitamin D-related genetic risk score on birth length: the Vitamin D Pregnant Mother (VDPM) cohort study

**DOI:** 10.1186/s12884-022-05020-3

**Published:** 2022-09-07

**Authors:** Arif Sabta Aji, Nur Indrawaty Lipoeto, Yusrawati Yusrawati, Safarina G. Malik, Nur Aini Kusmayanti, Isman Susanto, Siti Nurunniyah, Ratih Devi Alfiana, Wahyuningsih Wahyuningsih, Nur Mukhlishoh Majidah, Karani Santhanakrishnan Vimaleswaran

**Affiliations:** 1 Department of Nutrition, Faculty of Health Sciences, Alma Ata University, Bantul, 55183 Indonesia; 2 Graduate School of Public Health, Faculty of Health Sciences, Alma Ata University, Bantul, 55183 Indonesia; 3 Department of Nutrition, Faculty of Medicine, Andalas University, Padang, 25127 Indonesia; 4 Department of Obstetrics and Gynaecology, Faculty of Medicine, Andalas University, Padang, 25127 Indonesia; 5 Eijkman Institute for Molecular Biology, Jakarta, 10430 Indonesia; 6 Faculty of Public Health, Universitas Ahmad Dahlan, Yogyakarta, Indonesia; 7 Department of Midwifery, Faculty of Health Sciences, Alma Ata University, Bantul, 55183 Indonesia; 8 Department of Nursing, Faculty of Health Sciences, Alma Ata University, Bantul, 55183 Indonesia; 9 Department of Food and Nutritional Sciences, Hugh Sinclair Unit of Human Nutrition, University of Reading, Reading, UK; 10 Institute for Food, Nutrition, and Health, University of Reading, Reading, UK

**Keywords:** Vitamin D, IGF-1, Genetic risk score, Birth length, Carbohydrate intake, Newborn anthropometry

## Abstract

**Background:**

Our objectives were to investigate the relationship between maternal vitamin D status and IGF-1 levels in healthy Minangkabau pregnant mothers and their impact on newborn anthropometry outcomes and to examine whether this relationship was modified by dietary intake using a nutrigenetic approach.

**Methods:**

Healthy singleton pregnant mother and infant pairs (*n* = 183) were recruited. We created three genetic risk scores (GRSs): a six-SNP GRS based on six vitamin D-related single nucleotide polymorphisms (SNPs) involved in the synthesis of vitamin D (vitamin D-GRS), a two-SNP GRS using SNPs in *VDR* genes (*VDR*-GRS) and a four-SNP GRS using SNPs from *DHCR7*, *GC*, *CYP24A1* and *CYP2R1* genes (non-*VDR* GRS). The effect of the GRSs on IGF-1, vitamin D and newborn anthropometry and the interaction between the GRSs and dietary factors were tested using linear regression analysis.

**Results:**

The vitamin D- and non-*VDR* GRSs were significantly associated with lower 25(OH)D concentration (*p* = 0.005 and *p* = 0.001, respectively); however, there was no significant association with IGF-1, and newborn anthropometry outcomes. However, there was a significant interaction of *VDR*-GRS with carbohydrate intake on birth length outcome (P_*interaction*_ = 0.032). Pregnant mothers who had higher carbohydrate intake (405.88 ± 57.16 g/day) and who carried ≥ 2 risk alleles of *VDR*-GRS gave birth to babies with significantly lower birth lengths compared to babies born to mothers with < 2 risk alleles (*p* = 0.008).

**Conclusion:**

This study identified a novel interaction between *VDR*-GRS and carbohydrate intake on birth length outcome. These findings suggest that reducing the intake of carbohydrates during pregnancy, particularly for those who have a higher genetic susceptibility, might be an effective approach for preventing foetal growth abnormalities.

**Supplementary Information:**

The online version contains supplementary material available at 10.1186/s12884-022-05020-3.

## Background

The neonatal mortality rate in Indonesia in 2017 was reported to be 12.4 per 1,000 live births [1], which is higher than in other South East Asian countries [2]. Key factors in this high prevalence may include the poor quality of perinatal health services, newborns with low birth weight and smaller size, and short birth interval factors [3]. Optimal intrauterine growth is necessary for foetal development and contributes to the long-term health of the newborn. Foetal growth may also be influenced by the interactions between genetic, nutritional, hormonal and environmental factors [4].

Vitamin D deficiency is a worldwide public health concern [5] and is mainly caused by inadequate exposure to sunlight. Vitamin D deficiency has been recognised as an epidemic in many regions, including Europe, America, the Middle East and Asia [6–9]. Vitamin D status during pregnancy has a significant impact on maternal health and foetal growth [10]. Vitamin D is a potentially modifiable regulator of the Insulin-like Growth Factor 1 (IGF-1) axis and a positive correlation has been demonstrated between serum 25-hydroxyvitamin D (25(OH)D) and IGF-1 levels [11, 12]. Placental growth hormones (GH) are produced by the placental syncytiotrophoblast and gradually replace pituitary GH from eight weeks of gestation in maternal circulation, increasing during pregnancy. The increase in maternal serum IGF-1 is thought to be caused by placental GH [13]. Previous studies have also demonstrated that maternal serum IGF-1 levels are significantly associated with increasing gestational age [14, 15]. However, while recent cross-sectional studies based on prospective cohort design found a positive relationship between serum 25(OH)D and IGF-1 levels [11, 12, 16, 17], the relationship between vitamin D and IGF-1 levels in pregnancy outcomes remains unknown.

It has been determined that vitamin D-related single nucleotide polymorphisms (SNPs) affect 25(OH)D concentrations, yet only a few studies have found evidence of this in South East Asian populations, notably in Minangkabau mothers, West Sumatra, Indonesia [18–20]. The Minangkabau is a matrilineal community in West Sumatra that has a high prevalence of vitamin D deficiency [21–27], which comes despite the tropical climate and abundant sunlight exposure all year round in Indonesia. Low vitamin D status has been shown to have a negative impact on foetal growth and development in areas such as bone development and the immune and nervous systems during pregnancy [28, 29]. In addition to genetic factors, race and ethnicity play an important role in the determination of vitamin D status [30]. Dietary factors also contribute to vitamin D status [18, 28, 29].

To our knowledge, no prior study has examined the relationship between vitamin D, IGF-1 and newborn anthropometry in Indonesia, particularly among the Minangkabau population, West Sumatra. This study identified whether this relationship was modified by dietary intake during pregnancy using a nutrigenetic study. Due to the high level of confounding that can influence phenotypic associations, we created three genetic risk scores (GRSs) using genetic variants as markers of maternal vitamin D concentration, given that genetic associations are less prone to confounding, and tested for their association with 25(OH)D, IGF-1 and newborn anthropometry outcomes.

## Methods

### Study population

The Vitamin D Pregnant Mother (VDPM) cohort study was conducted in West Sumatra Province, Indonesia from 1 June 2017 to 1 May 2018. The study design had been published previously [18, 20, 25, 31–34]. Different geographical locations were used, including two cities in mountainous areas (Payakumbuh, Lima Puluh Kota) and three cities in coastal areas (Padang, Pariaman, Padang Pariaman). The target population included the first trimester of pregnancy and their newborns (*n* = 183, *p* < 0.05). We followed all subjects up to their delivery process to perform newborn anthropometry measurements (birth weight, birth length and head circumference).

The VDPM study included mothers who 1) visited a public health care centre during the first trimester of pregnancy (< 13 weeks), 2) were healthy based on a doctor’s examination and health history, and 3) were willing to participate in the study, sign an informed consent and follow the research procedures. The exclusion criteria were mothers with multiple pregnancies, preeclampsia, miscarriage or stillbirth, chronic illnesses such as diabetes, hypertension, cardiovascular disease or hypothyroidism, and those who were taking drugs that can interfere with vitamin D metabolism. Of a total of 239 mothers, 53 dropped out for various reasons, including pregnancy loss, change of residence, unwillingness to continue with the research, and inability to contact again. This left a total of 186 pregnant mothers who completed all requirements and attended follow-ups from T1 to delivery. After subsequently excluding three samples due to low DNA yield, 366 subjects (183 mother and infant pairs) were used for the present study to determine the association between GRS and 25(OH)D concentration in newborn anthropometry. Additionally, 180 subjects were used to determine the association between IGF-1 and 25(OH)D concentration on newborn anthropometry and pregnancy outcomes and whether this relationship was modified by genetic variants of vitamin D. The subject recruitment process is shown in our recently published article [18].

### Subjects’ characteristics

Maternal sociodemographic factors were recorded using structured questionnaires. Demographic data including age, age group, education and sun exposure status were collected. These data were prospectively collected from medical records or interviews with the participants. Sun exposure status was defined by the duration of sun exposure, which was calculated as the average time spent daily in the sun during the participants’ leisure and working time. Maternal anthropometric measurements included pre-conception body weight, height and pre-pregnancy body mass index (BMI). These data were collected by a trained nutritionist to increase the accuracy of the anthropometric data collection. Pre-pregnancy BMI was calculated based on the routine height measurements taken during clinic visits and pre-pregnancy body weight data were obtained during interviews through the maternal and child monitoring book. Maternal body weight was measured to the nearest 50 g using an electronic scale (Seca 815, Seca GmbH. Co. kg, Germany) and height was measured to the nearest millimetre using a stadiometer (Seca 217, Seca GmbH. Co. kg, Germany). The BMI calculation was based on the body weight (kg) divided by the square of body height (m) and we used the Asia–Pacific classification to define nutritional status [35].

### Biochemical measurements

To determine IGF-1 and 25-hydroxyvitamin D (25(OH)D) levels, blood samples (3 ml) were collected by a trained phlebotomist in the first and third trimesters for 25(OH)D and the third trimester for IGF-1. The blood samples were used to measure serum IGF-1 and 25(OH)D levels using enzyme-linked immunosorbent assays (ELISA) with an xMark Microplate Spectrophotometer (Bio-Rad Laboratories Inc, Hercules, California, USA) according to the manufacturer’s instructions. Serum concentrations of IGF-1 were assessed using ELISA kits from Bioassay Technology Laboratory (Shanghai, China). The serum IGF-1 levels ranged from 2.11 ng/mL to 347.46 ng/mL and assay sensitivity was 0.058 ng/mL. The intra-assay and inter-assay values were < 8% and < 10%, respectively. Serum levels of 25(OH)D were assessed using ELISA from Diagnostic Biochemistry Canada (DBC) 25-Hydroxyvitamin D ELISA kit (DBC, London, Ontario Canada). The assay has a sensitivity of 5.5 ng/ml and intra- and inter-assay coefficients of variation of 5% and 8.1%, respectively. Vitamin D status was defined as serum 25(OH)D < 12 ng/mL (vitamin D deficient), 12–19 ng/mL (vitamin D insufficient) and ≥ 20 ng/mL (vitamin D sufficient) according to Institute of Medicine (IOM) guidelines [5].

### Dietary intake assessment

A trained nutritionist was recruited to collect the dietary intake data. The pregnant mothers were asked about their third-trimester dietary intake status. Macronutrients such as carbohydrates, protein and fat intake were collected and analysed after the data collection as crude intake (g/day). Dietary data and intake during pregnancy were collected and assessed using a validated semi-quantitative food frequency questionnaire (SQ-FFQ) [26, 27]. All data provided by the participants were analysed and the nutritionist asked the mothers to think specifically about their dietary consumption during their third trimester of pregnancy.

### SNPs selection and genotyping analysis

We selected six candidate SNPs based on the following criteria: (1) biological significance in vitamin D synthesis, and metabolism; (2) SNPs with minor allele frequency of > 5%, (3) evidence of significant association with the risk of adverse pregnancy outcomes that determine newborn anthropometry [36–38], and (4) evidence of a significant association with 25(OH)D concentrations in previous genome-wide association studies and RCT studies [39–42]. The selected genes were *DHCR7* (rs12785878), *CYP2R1* (rs12794714), *GC* (rs2282679), *CYP24A1* (rs6013897) and *VDR* (rs2228570 and rs7975232).

Blood samples were collected from all the study subjects. Genomic DNA was isolated from peripheral blood leukocytes using the PureLink Genomic DNA Mini Kit (Invitrogen, Carlsbad, USA). DNA was also extracted from the whole blood using the PureLink Genomic DNA Mini Kit (Invitrogen, Carlsbad, CA, USA). The DNA concentration was determined using a NanoDrop spectrophotometer (Isogen Life Science, De Meern, the Netherlands). Genotyping was performed using the competitive allele-specific PCR-KASP assay at LGC Genomics, London, UK.

### Pregnancy outcomes

Gestational age (GA) at birth was calculated from the estimated GA obtained by obstetricians or midwives through an examination using either a transabdominal ultrasound or the date of last menstrual period in the absence of ultrasound at a maternal clinic or hospital. Infant birth weight, birth length and head circumference were recorded at birth using Seca mechanical measuring scales (Seca 803, Seca GmbH. Co. kg, Hamburg, Germany). We classified newborn anthropometry status according to the World Health Organization Child Growth Standards for head circumference-for-age (small head circumference, < 35 cm and normal head circumference, ≥ 35 cm), weight-for-age (low birth weight, < 2,500 g and normal birth weight ≥ 2,500 g) and length-for-age (short birth length, < 50 cm and normal birth length, ≥ 50 cm) [43]. Placental weight was recorded by the obstetrician who delivered the baby and categorised as normal when the weight was greater than or equal to 500 g. Assessment of neonatal Apgar scores was undertaken at minutes 5 and 10 after birth. A newborn with 5-min and 10-min Apgar scores of 7 to 10 was categorised as normal status; subsequent 5-min and 10-min scores of < 7 were categorised as a low Apgar score. In addition, small-for-gestational age (SGA) was calculated as the weight below the 10^th^ percentile for gestational age. Preterm birth was described as a delivery that occurred before the 37^th^ week of pregnancy. The mode of delivery was classified as vaginal birth or caesarean section.

### Data analysis

We used the SPSS statistical package (version 23; SPSS Inc., Chicago, IL, USA) for the statistical analysis. The results from the descriptive analyses are presented as means ± standard deviations (SD) for continuous variables and as percentages for categorical variables. All six genetic variants were in the Hardy–Weinberg equilibrium (HWE) (*p* > 0.05), which was tested using a chi-square test [20]. Pre-pregnancy BMI was defined according to the World Health Organization Asia Pacific Guidelines for Asians as non-obese (BMI < 25 kg/m^2^) and obese (BMI ≥ 25 kg/m^2^) [44]. Normality of variable distribution was verified using the Shapiro–Wilk test; any variables that were not normally distributed were log-transformed prior to the analysis, such as 25(OH)D (ng/mL) and IGF-1 (ng/mL) concentration. Details of the power calculation have previously been published elsewhere [18].

Given the lack of any pre-existing cut-off points for IGF-1 status, the variable was divided into tertiles. The lowest tertile group was classified as those who had log-transformed IGF-1 ≤ 1.20 ng/mL, the medium tertile group was categorised as those who had values from 1.20 to 1.34 ng/mL and the highest tertile group was classified as those with values ≥ 1.35 ng/mL. Multivariate linear regression models were constructed to examine the phenotypic and genetic associations in this study: 1) the association between maternal vitamin D status and IGF-1 levels using a two-tailed t-test analysis, 2) the association between serum IGF-1 levels and newborn anthropometry outcomes, and 3) the association of vitamin D-related GRSs with 25(OH)D concentration and IGF-1 level during pregnancy.

The following interactions were tested: 1) the interaction between GRSs and serum IGF-1 levels on newborn anthropometry outcomes; 2) the interaction between GRS and 25(OH)D T3 levels on IGF-1 T3 levels during pregnancy; 3) the interaction between GRS and dietary intake on newborn anthropometry outcomes. These interactions were tested using linear regression after adjusting for potential confounding factors such as age, pre-pregnancy BMI, total energy intake, vitamin D, GA at birth and gender of the infant, wherever appropriate. *P* < 0.05 was considered statistically significant.

The three GRSs were created by summing the risk alleles from five genes [39–42]. The ‘Vitamin D-GRS’ was created from the six SNPs, rs12785878 (*DHCR7*), rs12794714 (*CYP2R1*), rs2282679 (*GC*), rs6013897 (*CYP24A1*), and rs2228570 and rs7975232 (*VDR*), that play a role in the synthesis and metabolism of vitamin D. Two SNPs in *VDR* genes were included in the ‘*VDR*-GRS’ and four SNPs in genes encoding proteins involved in 25(OH)D synthesis and metabolism (*GC, CYP24A1, DHCR7*) were included in the ‘non-*VDR*-GRS’. This study distinguished between *VDR-GRS* and *Non-VDR-GRS* due to the *VDR* gene variants associated with adverse pregnancy outcomes [18]. Assessing the influence of *VDR* gene variants alone may be a key factor in determining the association between 25(OH)D concentration and newborn anthropometry outcomes.

## Results

### Characteristics of the study subjects

The characteristics of the study participants stratified by third trimester (T3) vitamin D status are shown in Table 1. This study found that deficiency-insufficiency vitamin D status was significantly more prevalent among mothers aged 20–30 years than sufficiency vitamin D status (*p* = 0.025). There was no association between vitamin D status and newborn anthropometry outcomes (*p* > 0.05). Mothers with a sufficient vitamin D status had statistically higher IGF-1 levels than mothers with a deficiency-insufficiency vitamin D status (*p* = 0.036). The change in 25(OH)D concentration was significantly higher for mothers in the ‘sufficiency’ vitamin D status group (*p* < 0.001).

**Table 1:** Characteristics of study subjects based on T3 vitamin D status

Variables	‘Deficiency-insufficiency’ VD status (*n* = 86) (47.0%)	‘Sufficiency’ VD status (*n* = 97) (53.0%)	*P* Value
**Demography**			
Age, years	28.92 (5.07)	30.28 (6.12)	0.101
Maternal age group			**0.025**
a. ≤ 20	1	5.8	
b. 21–30	60.2	43.0	
c. > 30	38.8	51.2	
Education			0.255
a. Primary	23.5	31.4	
b. Secondary	38.8	41.9	
c. Tertiary	37.8	26.7	
Sun exposure status per day			0.721
a. < 1 h	52.6	48.8	
b. ≥ 1 h	47.4	51.2	
**Maternal anthropometry**			
Pre-conception body weight, kg	54.56 (11.21)	55.71 (10.15)	0.469
Height, cm	154.73 (5.79)	153.85 (6.65)	0.341
Pre-pregnancy BMI, kg/m^2^	23.12 (4.46)	23.61 (4.35)	0.457
Pre-pregnancy BMI status			0.361
a. < 25 kg/m^2^	72.4	65.1	
b. ≥ 25 kg/m^2^	27.6	34.9	
**Newborn outcomes**			
Gestational age at birth, weeks	39.08 (1.81)	38.73 (1.94)	0.211
Infant gender			
a. Boy	51	60.5	
b. Girl	49	39.5	
Birthweight, g	3147.09 (458.73)	3244.90 (469.51)	0.156
Birth length, cm	48.53 (2.05)	48.59 (3.43)	0.893
Head circumference, cm	33.55 (1.89)	34.10 (2.97)	0.139
Biochemical Measurements			
IGF-1, ng/mL	20.74 (12.89)	32.21 (1.89)	**0.036**
Changes in 25(OH)D, ng/mL	1.52 (6.17)	14.12 (8.40)	** < 0.001**

Data are presented as percentages (%) for categorical data variables and mean and standard deviation [mean (SD)] for numeric data variables. Indicators of vitamin D status during pregnancy are based on the Institute of Medicine (IOM); sufficient (≥ 20 ng/mL), insufficient (12–19.00 ng/mL) and deficient (< 12 ng/mL). Changes in 25(OH)D levels during pregnancy are defined by 25(OH)D T3 – 25(OH)D T1

*IGF-1* Insulin-like growth factor 1, *25(OH)D* 25-hydroxyvitamin D, *T1* First trimester, *T3* Third trimester, *BMI* Body mass index

### Association between IGF-1 and newborn anthropometry and pregnancy outcomes

There was no statistically significant association between IGF-1 levels and newborn anthropometry outcomes such as birthweight, birth length and head circumference (*p* > 0.05 for all comparisons, Additional File 1). Furthermore, there was no significant association between the IGF-1 levels and pregnancy outcomes such as small-for-gestational age (SGA status of preterm birth, mode of delivery, placental weight, Apgar 5’ and Apgar 10’ (*p* > 0.05 for all comparisons, Additional File 1)).

### Association between GRSs and 25(OH)D concentration

There was a statistically significant association between the vitamin D-GRS and non-*VDR*-GRS and log-transformed 25(OH)D concentration in both the crude and adjusted models (*p* < 0.05, for all comparisons), where individuals carrying a greater number of risk alleles had a lower 25(OH)D concentration than those carrying a smaller number of risk alleles (Table 2). There was no association between *VDR*-GRS and 25(OH)D concentration (*p* > 0.05, for all comparisons).

**Table 2:** Association between GRS and serum 25(OH)D levels during T3 of pregnancy

**Variables**	**N**	**Log 25(OH)D (ng/mL)**^a^			**Log 25(OH)D (ng/mL)**^a^		
		**β**	**Mean (SE)**	***P* value**^‡^	**β**	**Mean (SE)**	***P* value**^†^
Vitamin D-GRS total score*							
** ≤ 3**	110	0.08	1.31 (0.02)	**0.010**	0.08	1.31 (0.02)	**0.009**
** ≥ 4**	73		1.23 (0.02)			1.23 (0.02)	
*VDR*-GRS score**							
** < 2**	102	0.04	1.30 (0.02)	0.241	0.03	1.29 (0.02)	0.334
** ≥ 2**	79		1.26 (0.02)			1.26 (0.02)	
Non-*VDR* GRS score***							
** < 3**	124	0.09	1.30. (0.02)	**0.009**	0.10	1.31 (0.02)	**0.003**
** ≥ 3**	54		1.213 (0.03)			1.21 (0.03)	

Data are presented as mean and standard error [mean (SE)]

*25(OH)D* 25-hydroxyvitamin D, *SE* Standard error, VDR Vitamin D receptor, *GRS* Genetic risk score

^‡^*P* values obtained from linear regression analysis with the crude model

^†^*P* values obtained from linear regression analysis adjusted for age, pre-pregnancy BMI, sun exposure status, vitamin D supplement and geographical status

^a^The analysis was performed on log-transformed variables

^*^All six SNPs in genes are involved in the synthesis and metabolism of vitamin D

^**^Two SNPs in *VDR* genes are included in the ‘*VDR*-GRS score’

^***^Four SNPs in the *DHCR7*, *GC*, *CYP24A1* and *CYP2R1* genes are included in the ‘*Non*-*VDR* GRS score’

### Interaction between GRSs and serum IGF-1 levels on newborn anthropometry outcomes

As shown in Additional File 2, there was no statistically significant interaction between GRSs and log-transformed IGF-1 for newborn anthropometry outcomes such as birthweight, birth length and head circumference (*p* > 0.05 for all comparisons).

### Interaction between GRS and 25(OH)D concentration on serum IGF-1 levels during pregnancy

No statistically significant interactions were found between GRSs and log-transformed 25(OH)D concentration on log-transformed IGF-1 levels during pregnancy (*p* > 0.05 for all comparisons) (Additional File 3).

### Interaction between GRS and dietary intake on newborn anthropometry outcomes

**Fig. 1:**
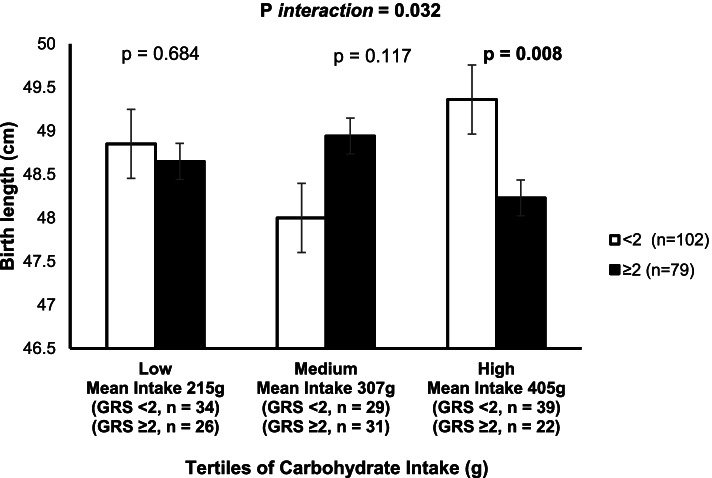
Interaction between the *VDR*-GRS and dietary carbohydrate intake (g) on birth length (cm) (P_*interaction*_ = 0.032). Mothers who were in the highest tertile of carbohydrate intake and carried ≥ 2 risk alleles gave birth to babies with significantly lower birth length (*p* = 0.008)

There was a statistically significant interaction between *VDR*-GRS and carbohydrate intake on birth length outcomes (P_*interaction*_ = 0.032). As shown in Fig. 1, those who were in the highest tertile of carbohydrate intake (mean ± SD: 405 ± 57.16 g/day) and carried ≥ 2 risk alleles gave birth to babies with a significantly lower birth length compared to babies from mothers carrying < 2 risk alleles (*p* = 0.008). None of the other interactions on newborn anthropometry outcomes was statistically significant (Table 3).

**Table 3:** Interaction between GRSs and T3 dietary intake on newborn anthropometry outcomes

**Genetic risk score (GRS)**	**Birth weight (g)**				**Birth length (cm)**			**Head circumference (cm)**		
	**N**	**Mean**	**Std. Error**	**P**_**interaction**_	**Mean**	**Std. Error**	**P interaction**	**Mean**	**Std. Error**	**P**_**interaction**_
Vitamin D-GRS*										
** ≤ 3 risk alleles**	110	3197.46	40.90	0.611^a^ 0.872^b^ 0.524^c^	48.75	0.19	0.065^a^ 0.073^b^ 0.300^c^	33.97	0.18	0.982^a^ 0.364^b^ 0.227^c^
** ≥ 4 risk alleles**	73	3233.58	50.84		48.70	0.23		33.93	0.23	
VDR-GRS**										
** < 2 risk alleles**	102	3188.11	43.30	0.810^a^ 0.775^b^ 0.556^c^	48.80	0.19	**0.032**^a^ 0.099^b^ 0.447^c^	34.01	0.19	0.970^a^ 0.701^b^ 0.571^c^
** ≥ 2 risk alleles**	79	3229.51	50.48		48.65	0.23		33.83	0.22	
Non-VDR GRS***										
** < 3 risk alleles**	124	3250.11	38.82	0.841^a^ 0.795^b^ 0.710^c^	48.79	0.18	0.256^a^ 0.079^b^ 0.278^c^	34.06	0.17	0.835^a^ 0.230^b^ 0.168^c^
** ≥ 3 risk alleles**	55	3148.72	60.40		48.56	0.28		33.80	0.26	

*IGF-1* Insulin-like growth factor 1, *PAL* Physical activity level, *T3* Third trimester

Adjusted for age, total energy intake in T3, pre-pregnancy BMI and vitamin D

^a^Interaction between GRS and dietary carbohydrate intake

^b^interaction between GRS and dietary protein intake

^c^Interaction between GRS and dietary fat intake

^*^All six SNPs in genes involved in the synthesis and metabolism of vitamin D (vitamin D-GRS);

^**^Two SNPs in *VDR* genes are included in the ‘*VDR*-GRS’;

^***^Four SNPs in *DHCR7*, *GC*, *CYP24A1* and *CYP2R1* genes are included in the ‘Non-*VDR* GRS score’

## Discussion

The current study is the first to investigate the interaction of maternal genetic susceptibility with dietary factors on maternal vitamin D and IGF-1 levels, and newborn anthropometric measurements in South East Asia. Using a genetic approach, our study has shown evidence for a novel interaction between *VDR*-GRS and carbohydrate intake on birth length outcomes, where mothers with higher *VDR* risk alleles and high carbohydrate consumption (339–581 g/day) gave birth to babies with lower birth length. The mean carbohydrate intake for the highest tertile was above the level stipulated in the Indonesian dietary guidelines [45]. Hence, these findings may have a significant public health implication in terms of improving newborn anthropometric measurements such as birth length by developing dietary intervention strategies to reduce carbohydrate intake for those who have higher vitamin D-related genetic risks.

Our study found an association between vitamin D status and IGF-1 levels but no significant association between IGF-1 levels and newborn anthropometric measurements and interaction of GRS with 25(OH)D, and IGF-1 on newborn anthropometric measurements, respectively, were observed. Our study findings are in accordance with other studies [12, 46]. A study of 241 healthy individuals also demonstrated a positive correlation between serum 25(OH)D and IGF-1 levels [11]. Previous studies have shown that IGF-1 stimulates the activity of enzyme 1α-hydroxylase, which has been shown to control the renal production of the active form of vitamin D [calcitriol or 1,25(OH)D] [47]. A previous intervention study in an Italian adult population showed that vitamin D supplementation increases the production of IGF-1 levels [12]. These results suggest that the association between vitamin D and foetal growth might depend on the effect of vitamin D on IGF-1 during pregnancy.

Even though GRS was not associated with newborn anthropometry, there was an interaction of *VDR*-GRS with carbohydrate intake on birth length. Those who consumed a high-carbohydrate diet and had higher risk alleles of vitamin D deficiency gave birth to babies with significantly lower birth lengths than those babies born to mothers with lower risk alleles. The average carbohydrate intake for the pregnant mothers in the highest tertile was 405.88 ± 57.16 g/day, which is above the Indonesian dietary recommendation [45, 48] of up to 360–400 g/day or equal to 50–60% of total energy percentage during the third trimester of pregnancy. A review of studies [48] from Hatriyanti et al. showed that no studies identified a deficiency in fat or carbohydrate intake for Indonesian pregnant mothers; in contrast, they mostly consume high amounts of dietary carbohydrates and fat during pregnancy. The Minangkabau Indonesia Study on Nutrition and Genetics (MINANG) also demonstrated that in the dietary intake of Indonesian Minangkabau women [19], more than 70% of the daily energy requirement was obtained from carbohydrates. This is not surprising because Indonesian people generally consume rice as a staple food and hence carbohydrate is the main source of energy during pregnancy [49]. Excess macronutrient intake, including high carbohydrate consumption during pregnancy, can increase maternal blood glucose levels and lead to pregnancy outcomes such as macrosomia [50].

The nutritional status of pregnant mothers is an important determinant in maintaining maternal health, such as preventing intrauterine growth restriction and neonatal size [51]. During pregnancy, vitamin D has been shown to be crucial for the development of the foetal skeletal system, immune system and tooth formation, along with the general growth of the foetus [21]. A study among West Javanese pregnant mothers revealed a negative association between low levels of vitamin D during pregnancy and SGA [52]. The prevalence of vitamin D deficiency has been reported to exceed 95% in the North Sumatra population [53] and 90% among pregnant mothers living in Jakarta [54, 55]. These studies demonstrate the fact that maternal vitamin D deficiency may be related to adverse pregnancy outcomes as well as newborn anthropometry. However, the results have been conflicting due to the small sample size in some studies, cross-sectional study design, lack of adjustment for seasonal variation, ethnicity, differences in study design in terms of the trimester (1^st^, 2^nd^ or 3^rd^ trimester), and the different cut-off points for categorising vitamin D status. Such inconsistencies highlight the need for large prospective and intervention studies to examine these causal associations.

Our study has been the first to investigate the interaction between maternal genetic susceptibility and dietary factors on newborn anthropometry in Indonesia. No published research exists on the interaction between vitamin D pathway-related SNPs and dietary factors on newborn anthropometry in Indonesia, although there have been limited studies in other countries. A study from a Japanese cohort comprising 78,793 paired mothers and their singleton offspring found that increased maternal carbohydrate intake was causally associated with increased birth length; however, the study did not explore the genetic susceptibility of the pregnant mothers [56]. In contrast, our study has shown that mothers with high carbohydrate intake gave birth to babies with lower birth length if they had a high *VDR*-GRS compared to mothers with a low *VDR*-GRS. While the relationship between *VDR* and foetal outcomes such as newborn anthropometry remains unclear, the role of *VDR* in normal and abnormal pregnancy conditions such as preeclampsia, foetal growth restriction, gestational diabetes and preterm birth have been reported in previous studies [57–59]. In addition, a recent meta-analysis [38] in 615 pregnant mothers showed that *VDR* genetic polymorphisms may play an important role in neonatal anthropometry via innate immunity and implantation, thereby suggesting that *VDR* could be a key factor in foetal growth and newborn anthropometry outcomes, making it a strong candidate gene for our study.

The mechanism by which carbohydrates interact with vitamin D pathway-related SNPs and affect newborn anthropometry, such as birth length outcome, is unclear and requires further investigation. The interaction of *VDR*-GRS with dietary carbohydrate intake during pregnancy on newborn anthropometry that was observed in our study is biologically plausible, given that vitamin D has been shown to regulate the development of metabolic diseases through its action on the metabolism of carbohydrates and its role in insulin secretion and sensitivity [60]. During pregnancy, the consumption of a high-carbohydrate diet can lead to excess glucose which is stored either as glycogen or converted into fatty acids and stored as fat in adipose tissue [61]. On the one hand, an excess of adipose tissue leads to its deposition and a decrease in vitamin D bioavailability; on the other hand, vitamin D deficiency in obesity affects the pathogenetic mechanisms associated with impaired tissue sensitivity to insulin and systemic inflammatory responses, promoting the development of insulin resistance and DM during pregnancy [62]. In addition, *VDR* and vitamin D-metabolising enzymes have been found to be strongly expressed in pancreatic beta cells and insulin-responsive cells such as adipocytes [63]. The polymorphisms of *VDR* may be associated with insulin resistance, which may lead to the risk of gestational diabetes mellitus (GDM) [64]. While a few studies have demonstrated a link between GDM and foetal macrosomia [65, 66], a recent meta-analysis found that specific patterns of *VDR* polymorphisms influence birth weight and other anthropometric neonatal outcomes [38]. High-risk alleles of vitamin D deficiency due to genetic susceptibility may lead to pregnancy complications and affect pregnancy outcomes such as lower birth length in the presence of a high-carbohydrate diet. An understanding of the genetic variants will yield positive results based on the implementation of personalised nutrition to prevent adverse pregnancy outcomes and maintain a balanced diet during pregnancy. Furthermore, future larger studies are required to confirm this finding.

The strengths of the current study include being the first nutrigenetic approach to determine the interaction between genetic variations and dietary factors on newborn anthropometry measurements among Minangkabau women. The construction of the GRSs, as opposed to a single SNP approach, improved the statistical ability to analyse the gene–nutrient interactions. Furthermore, we used a comprehensive, validated SQ-FFQ, which in turn enhanced the accuracy of the dietary data collection. In addition, the exposures examined in our study were collected by well-trained staff who followed validated and standard operating procedures. However, this study also has limitations that should be acknowledged. The main limitation of this study was its small sample size; however, the study was able to demonstrate significant associations and interactions. Although we used a validated SQ-FFQ, it is impossible to rule out bias due to the self-reported nature of the dietary intake information. The present study had no data on the specific categories of foods consumed during the dietary intake data collection, notably on the quantification of different types of carbohydrates into complex, simple and monosaccharides. The study participants were not screened for GDM and hence this could be a confounder in our study. Furthermore, the serum levels of 25(OH)D concentration were not measured by the liquid chromatography tandem mass spectrometry assay, which has been demonstrated to be the gold standard technique for the measurement of vitamin D metabolites. Finally, the study findings were limited to pregnant mothers among the Minangkabau people and thus cannot be generalised to the whole Indonesian population.

## Conclusions

The present study has demonstrated a novel interaction between *VDR*-GRS and carbohydrate intake on birth length outcomes among Indonesian pregnant Minangkabau mothers where individuals with a higher genetic risk of low vitamin D concentration and higher consumption of carbohydrates gave birth to babies with lower birth length. These findings are relevant for public health as they suggest the need for intervention to reduce the carbohydrate intake of Indonesian pregnant mothers given that a third of our study participants had a mean carbohydrate intake of ~ 405 g/day. This is equivalent to 66.48% of daily carbohydrate intake, based on the average carbohydrate intake (i.e. 2,441 kcal/day) in our study population, which exceeded the Indonesian dietary guidelines for pregnant mothers. During the third trimester, carbohydrates should account for 50–60% of daily total energy, which includes approximately 6–9 servings of whole grains daily [45]. Future studies with larger sample sizes and objective measures of carbohydrate intake, such as the type of carbohydrate, are needed to confirm these findings, which may be useful in establishing dietary interventions to overcome the genetic susceptibility of vitamin D deficiency and improve newborn outcomes.

## Supplementary Information

**Additional file 1.**

**Additional file 2.**

**Additional file 3.**

## Data Availability

The datasets generated and/or analysed during the current study are not publicly available due additional results from the study are yet to be published but are available from the corresponding author on reasonable request.

## Abbreviations

IGF-1: Insulin-like growth factor 1
VDPM: Vitamin D of pregnant mothers
MINANG: Minangkabau Indonesia study on nutrition and genetics
SNPs: Single nucleotide polymorphisms
GWAS: Genome-wide Association Studies
HWE: Hardy–Weinberg equilibrium
GRS: Genetic risk score
DHCR7: 7-Dehydrocholesterol reductase
CYP2R1: Cytochrome P450 Family 2
GC: Vitamin D binding protein
CYP24A1: Cytochrome P450 Family 24
VDR: Vitamin D receptor
25(OH)D: 25-Hydroxyvitamin D
1,25(OH)D: 1α,25-Dihydroxyvitamin D
BMI: Body mass index
GA: Gestational age
SGA: Small-for-gestational-age
T1: First trimester
T3: Third trimester
PTB: Preterm birth
