# Supplementary material for: Impact of maternal dietary carbohydrate intake and vitamin D-related genetic risk score on birth length: the Vitamin D Pregnant Mother (VDPM) cohort study
Source: BMC Pregnancy Childbirth. 2022 Sep 7;22:690. doi: 10.1186/s12884-022-05020-3 (PMC9450237; doi:10.1186/s12884-022-05020-3)
Supplement: Supplementary file 1 — Additional file 1. [file 12884_2022_5020_MOESM1_ESM.docx]

**Additional File 1.** Association between serum IGF-1 levels and newborn anthropometry, and pregnancy outcomes.

| Variables | IGF-1 tertiles | | | |
| --- | --- | --- | --- | --- |
|  | **Low**  **(n=60)** | **Medium**  **(n=60)** | **High**  **(n=60)** | **P value** |
| *Newborn anthropometry outcomes* |  |  |  |  |
| Birth weight, g | 3167.33 (55.36) | 3237.25 (55.74) | 3222.92 (55.90) | 0.643* |
| Birth length, cm | 48.74 (0.25) | 48.69 (0.26) | 48.74 (0.26) | 0.991* |
| Head circumference, cm | 33.72 (1.97) | 34.23 (1.89) | 33.95 (1.86) | 0.461* |
| *Pregnancy outcomes* |  |  |  |  |
| Preterm birth delivery status |  |  |  |  |
| 1. Normal (≥37 weeks) | 90.0 | 93.3 | 88.3 | 0.635^†^ |
| 1. Preterm (<37 weeks) | 10.0 | 6.7 | 11.7 |  |
| Mode of delivery |  |  |  |  |
| 1. Per vaginal | 66.7 | 76.7 | 76.7 | 0.360^†^ |
| 1. C section | 33.3 | 23.3 | 23.3 |  |
| Placental weight status |  |  |  |  |
| 1. Normal (≥500 g) | 86.7 | 85.0 | 85.0 | 0.956^†^ |
| 1. Low (<500 g) | 13.3 | 15.0 | 15.0 |  |
| Apgar 5' Status |  |  |  |  |
| 1. Normal | 85.0 | 99.3 | 88.3 | 0.819^†^ |
| 1. Low | 15.0 | 11.7 | 11.7 |  |
| Apgar 10' Status |  |  |  |  |
| 1. Normal | 91.7 | 93.3 | 95.0 | 0.765^†^ |
| 1. Low | 8.3 | 6.7 | 5.0 |  |

Data are presented as percentage (%) for categorical data variables and mean and standard error [mean (SE)] for numeric data variables.

AGA, appropriate for gestational age; SGA, small for gestational age.

*P values for newborn outcomes were obtained by using linear regression with adjusted for infant gender, maternal age, pre-pregnancy BMI, and gestational age at birth.

^†^P values for pregnancy outcomes were obtained by using chi square test.
