# Supplementary material for: Impact of maternal dietary carbohydrate intake and vitamin D-related genetic risk score on birth length: the Vitamin D Pregnant Mother (VDPM) cohort study
Source: BMC Pregnancy Childbirth. 2022 Sep 7;22:690. doi: 10.1186/s12884-022-05020-3 (PMC9450237; doi:10.1186/s12884-022-05020-3)
Supplement: Supplementary file 2 — Additional file 2. [file 12884_2022_5020_MOESM2_ESM.docx]

**Additional File 2.** Interaction between genetic risk score and Serum IGF-1 Levels on Newborn Anthropometry Outcomes.

|  | Birth weight (g) | | | | Birth length (cm) | | | | Head circumference (cm) | | | |  |
| --- | --- | --- | --- | --- | --- | --- | --- | --- | --- | --- | --- | --- | --- |
| *Vitamin D GRS** | | | | | | | | | | | | |  |
|  | **N** | **Mean** | **Std. Error** | **P interaction** | | **Mean** | **Std. Error** | **P interaction** | | **Mean** | **Std. Error** | **P interaction** | |
| Less than or equal 3 | 110 | 3210.00 | 45.39 | 0.874 | | 48.40 | 0.275 | 0.894 | | 33.96 | 0.191 | 0.782 | |
| Greater than or equal 4 | 70 | 3213.96 | 49.43 |  |  | 48.78 | 0.305 |  |  | 33.86 | 0.208 |  |  |
| *VDR GRS*** | | | | | | | | | | | | |  |
| Less than 2 | 102 | 3197.62 | 43.21 | 0.669 | | 48.79 | 0.20 | 0.684 | | 33.97 | 0.191 | 0.930 | |
| greater than or equal 2 | 76 | 3230.60 | 50.33 |  |  | 48.66 | 0.23 |  |  | 33.89 | 0.22 |  |  |
| *Non-VDR GRS**** | | | | | | | | | | | | |  |
| Less than 3 | 122 | 3226.77 | 39.38 | 0.910 | | 48.71 | 0.18 | 0.658 | | 34.01 | 0.17 | 0.889 | |
| Greater than or equal 3 | 54 | 3184.36 | 59.40 |  |  | 48.76 | 0.27 |  |  | 33.85 | 0.26 |  |  |

GRS, genetic risk score; IGF-1, Insulin-like growth factor 1. Adjusted for age, pre-pregnancy BMI, gestational age at birth, and infant gender.

*All six SNPs in genes involved in synthesis and metabolism of vitamin D

**Two SNPs in *VDR* genes included in the “*VDR* GRS score”

***Four SNPs in *DHCR7, GC, CYP24A1*, and *CYP2R1* genes are included in the “Non-*VDR* GRS score”
