# Supplementary material for: Impact of maternal dietary carbohydrate intake and vitamin D-related genetic risk score on birth length: the Vitamin D Pregnant Mother (VDPM) cohort study
Source: BMC Pregnancy Childbirth. 2022 Sep 7;22:690. doi: 10.1186/s12884-022-05020-3 (PMC9450237; doi:10.1186/s12884-022-05020-3)
Supplement: Supplementary file 3 — Additional file 3. [file 12884_2022_5020_MOESM3_ESM.docx]

**Additional File 3.** Interaction between genetic risk score and 25(OH)D Concentration on Serum IGF-1 Levels during Pregnancy.

| Variables | IGF-1 (ng/mL) | | | |
| --- | --- | --- | --- | --- |
|  | **N** | **Mean** | **Std. Error** | **P interaction^a^** |
| Vitamin D-GRS total score* |  |  |  |  |
| Less than or equal 3 | 110 | 1.29 | 0.03 | 0.207 |
| Greater than or equal 4 | 70 | 1.30 | 0.03 |  |
| VDR GRS score** |  |  |  |  |
| Less than 2 | 102 | 1.27 | 0.03 | 0.547 |
| Greater than or equal 2 | 76 | 1.32 | 0.03 |  |
| Non-VDR GRS score*** |  |  |  |  |
| Less than 3 | 122 | 1.30 | 0.02 | 0.338 |
| Greater than or equal 3 | 54 | 1.30 | 0.04 |  |

GRS, genetic risk score; IGF-1, Insulin-like growth factor 1; 25(OH)D, 25-hydroxyvitamin D.

Adjusted for age and pre-pregnancy BMI.

*All six SNPs in genes involved in synthesis and metabolism of vitamin D

**Two SNPs in *VDR* genes included in the “*VDR* GRS score”

***Four SNPs in *DHCR7, GC, CYP24A1*, and *CYP2R1* genes are included in the “Non-*VDR* GRS score”

^a^The analysis was performed on log-transformed variables
